# Biology of Infantile Hemangioma

**DOI:** 10.3389/fsurg.2014.00038

**Published:** 2014-09-25

**Authors:** Tinte Itinteang, Aaron H. J. Withers, Paul F. Davis, Swee T. Tan

**Affiliations:** ^1^Gillies McIndoe Research Institute, Wellington, New Zealand; ^2^Centre for the Study and Treatment of Vascular Birthmarks, Wellington Regional Plastic, Maxillofacial and Burns Unit, Hutt Hospital, Wellington, New Zealand

**Keywords:** infantile hemangioma, renin–angiotensin system, beta-blocker, angiotensin-converting enzyme inhibitor, propranolol, captopril, hemogenic endothelium, placenta

## Abstract

Infantile hemangioma (IH), the most common tumor of infancy, is characterized by an initial proliferation during infancy followed by spontaneous involution over the next 5–10 years, often leaving a fibro-fatty residuum. IH is traditionally considered a tumor of the microvasculature. However, recent data show the critical role of stem cells in the biology of IH with emerging evidence suggesting an embryonic developmental anomaly due to aberrant proliferation and differentiation of a hemogenic endothelium with a neural crest phenotype that possesses the capacity for endothelial, hematopoietic, mesenchymal, and neuronal differentiation. Current evidence suggests a putative placental chorionic mesenchymal core cell embolic origin of IH during the first trimester. This review outlines the emerging role of stem cells and their interplay with the cytokine niche that promotes a post-natal environment conducive for vasculogenesis involving VEGFR-2 and its ligand VEGF-A and the IGF-2 ligand in promoting cellular proliferation, and the TRAIL-OPG anti-apoptotic pathway in preventing cellular apoptosis in IH. The discovery of the role of the renin–angiotensin system in the biology of IH provides a plausible explanation for the programed biologic behavior and the β-blocker-induced accelerated involution of this enigmatic condition. This crucially involves the vasoactive peptide, angiotensin II, that promotes cellular proliferation in IH predominantly via its action on the ATIIR2 isoform. The role of the RAS in the biology of IH is further supported by the effect of captopril, an ACE inhibitor, in inducing accelerated involution of IH. The discovery of the critical role of RAS in IH represents a novel and fascinating paradigm shift in the understanding of human development, IH, and other tumors in general.

## Introduction

**Figure 1:**
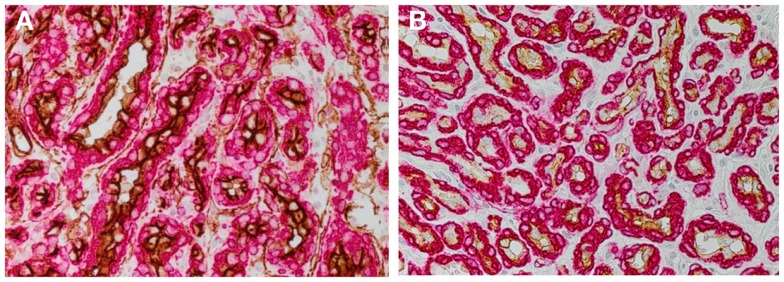
**DAB staining of proliferating infantile hemangioma showing the abundance of microvessels with an inner endothelium expressing the endothelial marker, CD34 [(A) brown], and an outer pericyte layer expressing smooth muscle actin [(A,B) red]**. The inner endothelial layer also expresses GLUT-1 [**(B)** brown], the immunohistochemical marker for infantile hemangioma. Cell nuclei are counterstained with hematoxylin [**(A,B)** blue]. Original magnification 400×.

Infantile hemangioma (IH) affects 4–10% of infants with a predilection for female, Caucasian, low birth weight, and premature infants (1–3). A higher incidence of IH has also been observed following amniocentesis (4), chorionic villous sampling (CVS) (5), and pre-eclampsia (6). IH has traditionally been regarded as a tumor of the microvasculature characterized by the proliferation of immature endothelial cells (ECs) (7) (Figure 1A). The expression of stem cell markers in IH has led to the notion that stem cells are recruited into the lesion (8, 9). However, recent data demonstrating a primitive signature on the endothelium of proliferating IH has shed new light on the biology of this tumor. This, along with the appreciation of the role of various cytokine signaling pathways in IH, has resulted in a paradigm shift in the understanding of this enigmatic condition.

This review outlines recent data pointing to a placental embolic origin of IH during early fetal life (10) and a post-natal conducive environment that supports proliferation and differentiation of the embolized primitive cells. The co-expression of multiple primitive markers in IH suggests an aberrant embryonic developmental anomalous nature of this condition (11) with evidence of a hemogenic endothelium (12) that possesses the capability for neuronal (13), mesenchymal (9, 14), endothelial (13), and hematopoietic (15) differentiation. Insights into the niche-environment within IH that regulates this primitive endothelium including the vascular endothelial growth factor (VEGF) system, the insulin-like growth factor (IGF) system, the TRAIL-osteoprotegerin (OPG) anti-apoptotic pathway, and the renin–angiotensin system (RAS), provide clues into the post-natal environment that modulates the programed biologic behavior of IH and underscores current and future novel therapies.

## Placenta and Infantile Hemangioma

### Placental antigens

The expression of glucose transporter-1 (GLUT-1) protein on the endothelium of IH (Figure 1B) was first reported in 2000 (16). The demonstration of GLUT-1 in IH and the placental syncytiotrophoblast microvilli and basement membranes (17), and the unique co-expression of the placental antigens, merosin, Lewis Y antigen, FCγRIII, and type 3-iodothyronine deiodinase (18–20), has led to the speculation of a placental embolic origin of IH.

### Placental cells

**Figure 2:**
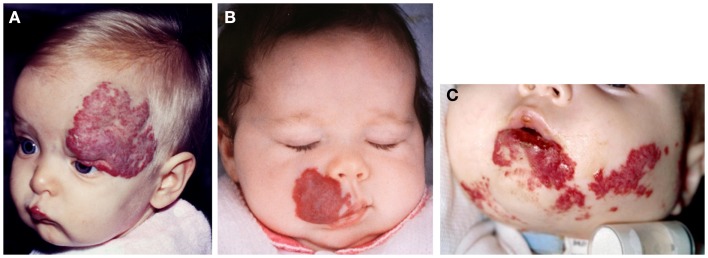
**Segmental infantile hemangioma in the “fronto-nasal” (A), “maxillary” (B), and “mandibular” (C) distribution**.

**Figure 3:**
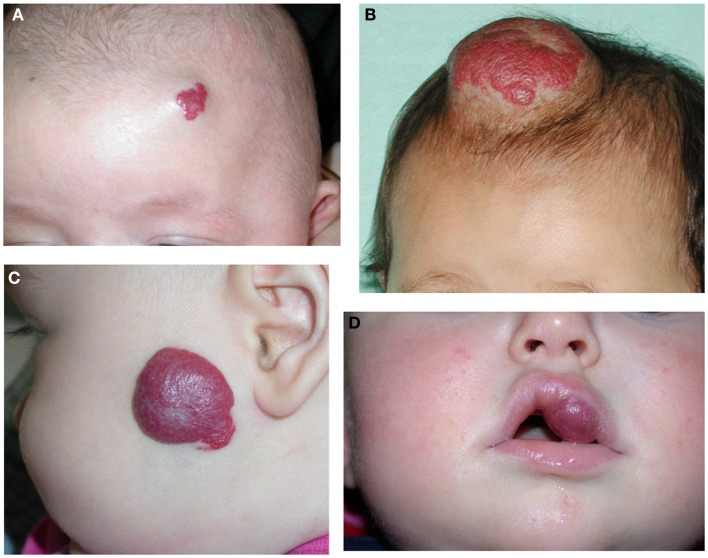
**A discrete proliferating infantile hemangioma on the forehead (A), scalp (B), cheek (C), and upper lip (D) of affected infants**.

Earlier work by Bree et al. (21) investigating the expression of the markers of placental trophoblasts, alkaline phosphatase, human placental lactogen (hPL), and cytokeratins 7 and 8, shows that none of these markers are expressed in IH. We have subsequently demonstrated the unique co-expression of human chorionic gonadotropin (hCG) and hPL, but not cytokeratin 7 and human leukocyte antigen-G (HLA-G), on the endothelium of proliferating IH (10) – a phenotypic signature of placental chorionic villous mesenchymal core cells (PCVMCCs). This led us to hypothesize a PCVMCC origin of IH and propose that these cells are embolized to the developing fetus proper in which they become embedded and integrated (10). We further hypothesized that embolization of PCVMCCs within the first trimester coinciding with the first migration of neural crest cells along their somitic routes (10) that occurs within the first month of gestation (22), leads to integration of these cells along the neural crest migratory routes manifesting as segmental lesions (Figure 2), while embolization later in gestation results in discrete lesion(s) (Figure 3) (10).

This hypothesis, if proven, would account for the increased incidence of IH in infants born following amniocentesis and CVS, with a higher incidence in CVS (4, 5). Amniocentesis and CVS are typically performed at 15 and 11 weeks of gestation, respectively (4), suggesting that earlier placental intervention is more likely to result in embolization of the PCVMCCs. A Cochrane review shows increased incidence of discrete IH lesions following amniocentesis and CVS (23), which are performed after the wave of neural crest migration that typically occurs within the first month of gestation (22).

## Stem Cells in Infantile Hemangioma

### Embryonic-like stem cells

The presence of embryonic stem cell (ESC) markers during the proliferative phase of IH with reduced expression as the lesion involutes, has been observed by several research groups (24, 25). The expression of ESC markers, such as Oct-4, SSEA-4, and STATS-3, has been demonstrated on the endothelium of proliferating IH (25). Interestingly, another population of cells expressing the ESC markers, Nanog (25, 26), SALL4 (24), and CD133 (24), has been localized in the interstitium of IH. More recent work has confirmed the presence of these markers within proliferating IH at the transcriptional level (27). It remains to be determined whether one set of cells gives rise to the other, or whether there are two distinct populations of embryonic-like stem cells within proliferating IH. However, the observation of co-expression of tryptase and Nanog in an interstitial cell population in proliferating IH, suggests that these interstitial cells represent a primitive myeloid phenotype, which we propose to have arisen from the putative hemogenic endothelium of IH (28). The exact role for these primitive myeloid cells with a mast cell phenotype is the focus of our ongoing research.

### Neural crest cells

**Figure 4:**
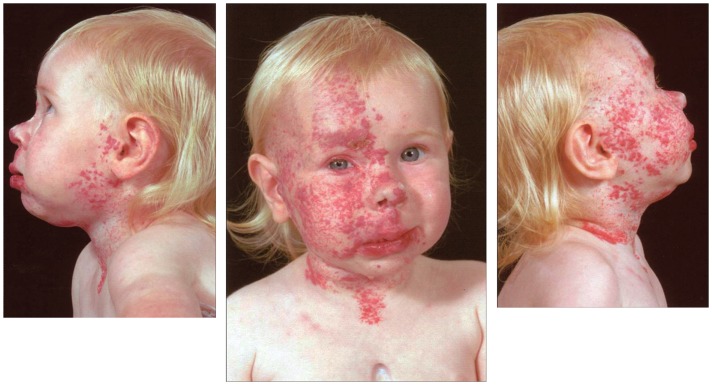
**A girl with segmental cervico-facial infantile hemangioma associated with a sternal cleft constituting PHACES syndrome**. Reproduced with permission from the Journal of Clinical Pathology (32).

Infantile hemangioma commonly presents as a discrete lesion(s) (Figure 3). A sub-group of IHs present as plaque-like lesions, distributed in a segmental manner (29) (Figure 2), forming a pattern similar to the meso-ectodermal fusion lines, along the neural crest somite migratory routes (30). The observation that some segmental IHs are associated with midline structural anomalies, constituting PHACES syndrome (31) (Figure 4), has led us to investigate the involvement of neural crest cells (32). We have shown the expression of the neural crest markers, p75, SOX-9, and SOX-10, on the endothelium of proliferating IH, thus displaying a neural crest phenotype (32). Interestingly, neural crest cells are the only cells that are developmentally capable of forming both mesenchymal as well as ectodermal lineage tissues (33). The co-expression of neural crest and ESC markers in the endothelium of proliferating IH, however, suggests that cells with neural crest phenotype are downstream to cells expressing ESC markers (34). Khan et al. (13) have demonstrated the ability of IH-derived cells to undergo neuronal differentiation, a downstream ectodermal lineage. Similarly, these IH-derived cells possess the potential for mesenchymal differentiation (9, 14).

### Primitive endothelial phenotype

Our investigations show that the endothelium of proliferating IH expresses a number of primitive lineage markers including the primitive mesoderm marker (brachyury) (12), mesenchymal markers (vimentin, CD29 and Pref-1) (14), and hematopoietic markers [ACE, GATA-2, Tal-1, and hemoglobin ζ (HBZ)] (12, 15, 35, 36). This unique co-expression pattern suggests that the endothelium of proliferating IH possesses a primitive phenotype with a hemogenic endothelium signature (37, 38), and also the ability for cells derived from proliferating IH to form mature ECs (13). The presence of an endothelial progenitor cell (EPC) population within proliferating IH lesions (8) and the circulation of affected patients (39), led to the earlier hypothesis that these cells are the origin of IH. However, the demonstration of a more primitive phenotype of the endothelium of proliferating IH, suggests that the circulating EPCs represent a more downstream population, rather than being the origin of IH (12).

### Hematopoietic differentiation

The demonstration of a hemogenic endothelium phenotype within proliferating IH has led us to investigate the functional capability of IH to undergo hematopoietic differentiation. We have shown that proliferating IH explant-derived cells possess the capability to undergo spontaneous erythropoiesis *in vitro* (15), with evidence of the presence of intermediate blast colonies characterized by the expression of VEGFR-2, CD34, CD133, and ACE (15, 25). We have further demonstrated the expression of the fetal hemoglobin, HBZ chain, and erythropoietin receptor, on the endothelium of proliferating IH (15). This, along with the functional ability of proliferating IH-derived cells to form erythrocytes expressing HBZ, suggests the capability of the endothelium of proliferating IH to undergo primitive erythropoiesis (15, 40). A recent report by Doege et al. (41) shows exogenous erythropoietin administration as an independent risk factor for developing IH, in pre-term infants. This observation implies the existence of the putative stem cell origin for IH, and the administration of erythropoietin creates an environment conducive for the development of IH.

### Mesenchymal differentiation

A mesenchymal progenitor cell (MPC) population within IH has been proposed to give rise to the fibro-fatty deposition that occurs during spontaneous involution of IH (9). These MPCs have been assumed to be recruited into the IH lesion, from either local niches or the bone marrow (9). However, the expression of the pre-adipocyte marker, Pref-1, on the endothelium of proliferating IH points to the phenotypic hemogenic endothelium being the origin of these MPCs (14, 42). The notion of an endothelial phenotype giving rise to mesenchymal progenitors has been previously reported for a CD34^+^ population (43), although the authors highlighted that the CD34^+^ cells were also CD31^−^, which taken in context, highlights CD34 as a primitive marker for both hematopoietic and endothelial (44) progenitors, as well as MPCs. The ability for IH-derived cells to undergo terminal mesenchymal differentiation (9, 14) confirms the existence of an MPC population within proliferating IH, potentially giving rise to the fibro-fatty residuum of involuted lesions.

## Cytokine Niche

### The vascular endothelial growth factor system

Vascular endothelial growth factor has been implicated in the proliferation of IH (45) with the demonstration of the local production of VEGF by the endothelium of IH (46). The VEGF-A isoform has been suggested to play a key role in the biology of IH, along with other hypoxia-induced factors that are up-regulated during proliferation of IH, by promoting an environment conducive for post-natal vasculogenesis (47).

Despite the inferred role of VEGF-A in IH, it was not until work by Jinnin et al. (48) that the demonstration of the VEGFR-2 isoform, coupled with the reduced decoy receptor VEGFR-1, led to the proposed predominant action of the VEGF-A ligand in promoting enhanced signaling via VEGFR-2 phosphorylation (48, 49) possibly with the aid of the molecular chaperone, COSMC (50). The expression of VEGF-A during proliferation of IH has more recently been localized to cells away from the endothelium (51), with corticosteroids inhibiting the effect on the vasculogenic potential of IH-derived stem cells, via reduction in VEGF-A expression (51).

### The insulin-like growth factor system

The insulin-like growth factors, IGF-1 and IGF-2, share a 60% homology in their amino acid sequences (52). The receptors for these cytokines are predominantly IGF receptors 1 and 2, differing by either the presence or absence of the β or α subunits, respectively (53, 54). Interestingly IGF-2 has been reported to be predominantly produced by proliferating IH, decreasing as the lesion involutes (42). Furthermore, the expression of IGF-2 has been localized to the endothelium of IH (42, 55). Further evidence using our *in vitro* explant model of IH (56) shows the effect of exogenous IGF-2 on stimulating outgrowths of cells from IH (42). Despite the increased knowledge about the role of IGF-2 in the biology of IH, there is currently no data on the expression of either of the two IGF receptors within IH. This is potentially an important pathway to investigate as IGF-2 is also known to bind to both insulin receptor (IR) isoforms A and B (53), with the IR-A isoform being predominantly expressed during human fetal development. It may be possible that IGF-2 is the ligand for the IGF receptors and the IRs in IH, with IH being an embryonic developmental anomaly (15, 57).

### The TRAIL-osteoprotegerin anti-apoptotic pathway

Osteoprotegerin, a soluble decoy death receptor for tumor necrosis factor-related apoptosis-inducing ligand (TRAIL), has recently been localized to the endothelium of IH and is up-regulated in proliferating lesions (58). Furthermore, the expression of TRAIL and its receptors, death receptors 4 and 5 and decoy death receptors 1 and 2, has been demonstrated at steady levels throughout the three phases of IH development (58). OPG is a pro-tumor survival factor (59) and its reduced levels during involution of IH (58) may account for the reduction in cellular density typically seen in involuted lesions (60), through TRAIL-mediated apoptosis. This is intriguing as clusterin/ApoJ, a glycoprotein that has been shown to be up-regulated during involution of IH (60), stimulates tumor necrosis factor-α (TNF-α) (61), a ligand for TRAIL, in association with cellular apoptosis (62). Therefore, OPG is implicated in anti-apoptosis during the proliferative phase of IH with the development of a putative environment conducive for cellular senescence through the indirect up-regulation of TNF-α, by clusterin/ApoJ (61), coupled with the decreased production of OPG (58).

### The renin–angiotensin system

**Figure 5:**
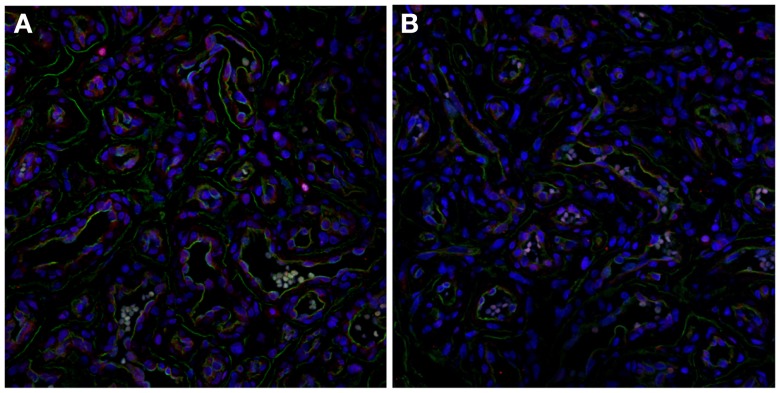
**Immunofluorescent staining of proliferating infantile hemangioma demonstrating the endothelium, with CD34 (green, A & B), also expressing ACE (A, red) and ATIIR2 isoform (B, red)**. Original magnification: 400x.

The role of the RAS in the biology of IH has been recently demonstrated by the expression of angiotensin-converting enzyme (ACE) (Figure 5A) and the angiotensin II receptor 2 (ATIIR2) (Figure 5B) isoform; the effect of angiotensin II (ATII) in inducing IH-derived blast cell proliferation (25); and the promotion of cellular proliferation in IH by ATII via the activation of ATIIR2, *in vitro* (63).

Serum levels of renin are approximately five folds that of adults within the first 3 months of life, tapering to three times that of the adult levels at 3–12 months of age, twice that of the adult levels at 1–4 years of age, with gradual reduction to normal adult levels from 8 years of age (64). The tapering levels of renin from birth through infancy and childhood (64) mirror the programed growth pattern for IH (65).

Renin levels have also been shown to be physiologically higher in female (compared with male), Caucasian (compared with black), and premature (compared with full-term) infants (66). This may account for the higher incidence of IH in these demographic groups (6).

**Figure 6:**
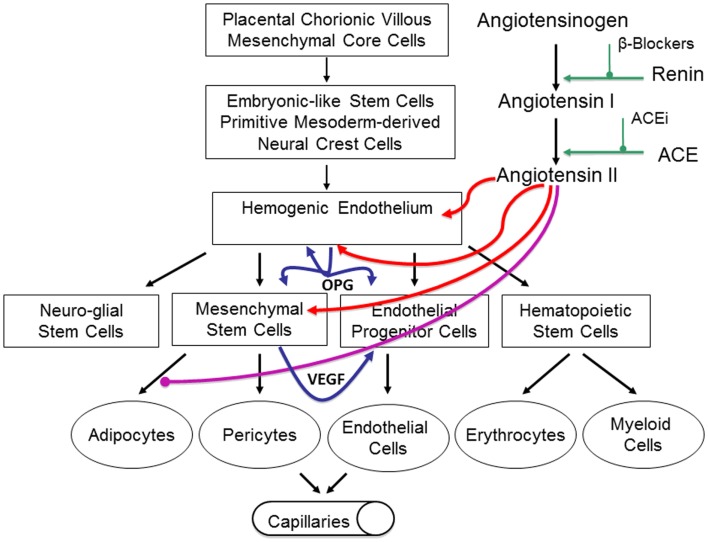
**Our proposed model of infantile hemangioma (IH) accounting for the observed programed biologic behavior and accelerated involution induced by modulators of the RAS, β-blockers, or ACE inhibitors**. IH is caused by aberrantly displaced/embolized placental chorionic villous mensenchymal core cells into the fetus proper, which gives rise to a primitive mesoderm-derived hemogenic endothelium with a neural crest phenotype regulated by the RAS. This hemogenic endothelium differentiates into stem cells of neuro-glial, mesenchymal, endothelial, and hematopoietic lineages with downstream mesenchymal and erythropoetic and potentially myeloid differentiation capabilities. During the proliferative phase of IH, high levels of renin indirectly lead to high levels of ATII resulting in aberrant proliferation of the hemogenic endothelium and secretion of vascular endothelial growth factor (VEGF) from the accumulating mesenchymal stem cells (MSCs), both leading to proliferation of the endothelial progenitor cells (EPCs) and downstream endothelial cells (ECs). High levels of ATII also lead to over-expression of the TRAIL decoy receptor, osteoprotogerin (OPG) preventing apoptosis of the hemogenic endothelium, MSCs, and EPCs, with further proliferation and accumulation of these cellular elements and ECs. High levels of ATII also prevent terminal differentiation of MSCs to downstream adipocytes, further increasing the accumulation of MSCs. During the involuting phase of IH, reduced levels of ATII indirectly caused by decreasing levels of renin, ease accumulation of EPCs and ECs. Reduced levels of ATII also allow termination differentiation of MSCs into adipocytes resulting in a fibro-fatty residuum. Inhibition of renin by β-blockers or ACEi leads to reduced levels of ATII resulting in accelerated involution of IH. Reproduced with permission from Plastic and Reconstructive Surgery (67).

High levels of renin, which convert the precursor angiotensinogen into angiotensin I, indirectly lead to high levels of ATII within IH that promotes a niche conducive for cellular proliferation (67). We have proposed the role of RAS in spontaneous and RAS modulator-induced accelerated involution of IH via its influence on the levels of both VEGF and OPG (67), although these remain the topic of our ongoing research. Based on previous reports, we have hypothesized that ATII promotes both VEGF and OPG secretion and maintain an environment favorable for vasculogenesis and anti-apoptosis (66) (Figure 6). However, these remain to be conclusively determined.

## The Renin–Angiotensin System and Novel Therapies

### β-blockers

The β-blockers, propranolol (68) and acetabutolol (69) were serendipitously discovered in 2008, by two independent French groups to cause accelerated involution of IH. Propranolol is now the preferred treatment for problematic proliferating IH (70, 71). Subsequently other β-blockers including timolol (72), nadolol (73), and atenolol (74) have also been described in the treatment of IH.

Various hypotheses have been advanced to account for the observed β-blocker-induced accelerated involution of IH (75) including vasoconstriction (71), decreased levels of VEGF, and fibroblast growth factor-2, leading to inhibition of angiogenesis (76) and the induction of apoptosis in proliferating ECs (77).

β-adrenergic receptors are expressed throughout the body with β1-receptors predominantly located in the heart and kidney, and β2-receptors predominantly located in peripheral blood vessels, skeletal muscle, and the lungs (78). In the kidney, β-adrenoreceptor blockade leads to inhibition of renin release and consequently results in modulation of the RAS (79).

The capability of β1 and non-selective β-blockers to induce accelerated involution of IH implies the common action of this class of drugs, via their modulation of RAS, rather than their inhibitory effects on the sympathetic nervous system or circulating catecholamines via β-adrenoreceptors blockade.

### β-blockers currently in use for the treatment of infantile hemangioma

**Figure 7:**
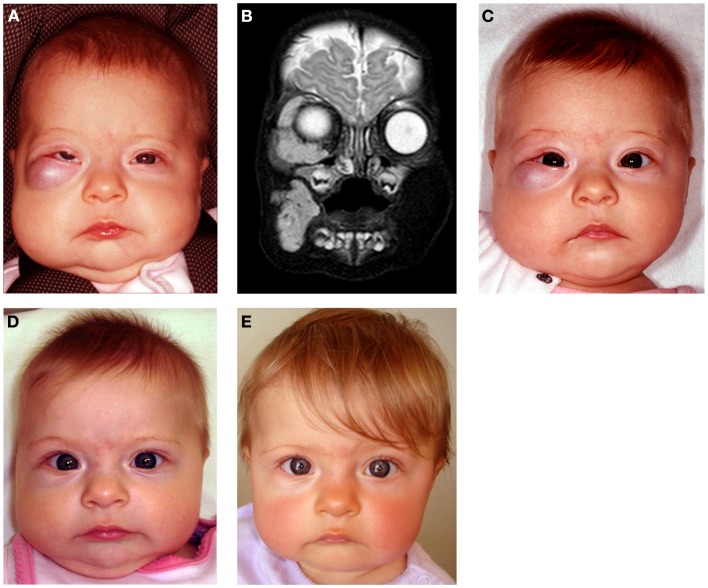
A 4-month-old girl presented with a rapidly growing infantile hemangioma on the right cheek, lower lid, and orbit with ocular dystopia **(A)** shown on a T2-weighted MRI scan **(B)**. Accelerated involution of the lesion with equalization of the globe 7 days **(C)**, 4 weeks **(D)**, and 5 months **(E)** following institution of propranolol therapy at 2 mg/kg/day. Reproduced with permission from Plastic and Reconstructive Surgery (70).

Propranolol, a non-selective β-blocker, is now the preferred treatment for problematic proliferating IH at a dosage of 2–3 mg/kg/day (80). However, the optimal escalation regimen, dosage, and treatment duration remain unknown. We have demonstrated that low-dose propranolol at 1.5–2.0 mg/kg/day is efficacious and has minimal complications (80) (Figure 7).

Acebutolol, a selective β1-blocker, is administered at a dosage of 8–10 mg/kg/day, with a clinical response generally observed 1 month after initiation of the treatment (81). Comparison of the efficacy of propanolol and acebutolol is currently the subject of a trial at the University Hospital at Montpellier, registered with the Clinical Trials US National Institute of Health.

Atenolol shows promise as an alternative to propranolol for IH. It is administered as a once daily dosing regimen of 1 mg/kg/day (82). As atenolol is a cardio-selective β-blocker, there is theoretically a reduced risk of adverse respiratory effects, which is beneficial in patients prone to bronchospasm whilst on propranolol (83).

Nadolol, a synthetic non-specific β-receptor antagonist, has been advocated for the treatment of IH as it is thought to have a better safety profile and greater compliance (its longer half-life allows for less frequent dosing) compared to propranolol (73). Nadolol is commenced at 0.5 mg/kg/day in two divided doses. The dosage is increased according to response on a weekly basis by 0.5 mg/kg up to a maximum of 4 mg/kg/day. As nadalol does not cross the blood brain barrier, there is a potentially reduced risk of nightmares and long-term memory loss (73).

Timolol, a topical non-selective β-blocker, administered as 0.5% timolol maleate ophthalmic solution (two drops per dose) (72) or as a gel preparation applied twice daily directly onto the IH (84), has been described with promising results in superficial lesions.

### Side effects of β-blockers in the treatment of infantile hemangioma

The side effects of β-blockers in the treatment of IH include hypotension, bradycardia, hypoglycemia, bronchospasm, hyperkalemia, nightmares, and gastrointestinal upsets (70). Propranolol, at 2–3 mg/kg/day for the treatment for proliferating IH, is associated with variable complication rates up to 61.2% (85). A meta-analysis of 41 reports with a mean dosage of 2.12 mg/kg/day shows complications occurring in 31% of patients (86). We (80) have previously shown that accelerated involution of IH occurs at a lower dosage of propranolol than that currently used. An individual patient’s minimal therapeutic dosage to treatment response can be determined by using a stepwise escalation regimen, thereby reducing side effects and treatment costs while improving outcomes. We have confirmed that propranolol, at 1.5–2 mg/kg/day, administered in divided doses is effective for treating proliferating IH with minor complications of 6.8% (87). We have also shown that treatment needs to continue to an average age of 14.2 months to avoid rebound growth (80, 87), reflecting the rapid tapering of circulating renin levels at that age (64).

### ACE inhibitors

**Figure 8:**
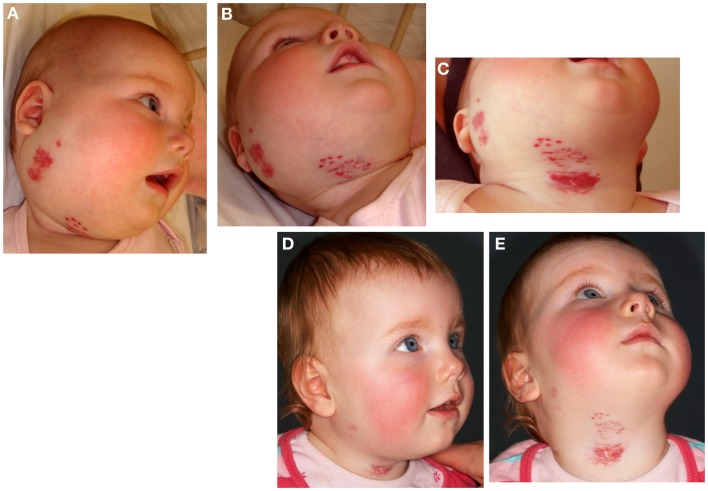
**A 22-week-old girl with a 7 cm × 10 cm proliferating infantile hemangioma in the right cervico-facial area causing significant tissue distortion before (A,B), 3 weeks (C), and 6 months (D,E) after administration of captopril at 1.5 mg/kg/day resulting in accelerated involution**. Reproduced with permission from British Journal of Dermatology (66).

Confirmation of the role of the RAS in the biology of IH and the observed effect of β-blockade has been supported by a prospective observational clinical study using captopril, an ACE inhibitor (66) (Figure 8). Treatment of IH with low-dose captopril at 1.5 mg/kg/day (compared to the cardiovascular dosage of up to 4 mg/kg/day) resulted in accelerated involution of IH in seven out of the eight subjects, with a more gradual response in the remaining patient (66). However, a retrospective review of IH patients treated with captopril for steroid-induced cardiomyopathy by Christou et al. (88) did not show any favorable results, although the dosage and duration of captopril administration was not reported.

## Conclusion

Infantile hemangioma is traditionally considered a tumor of the microvasculature (2, 7). However, recent data shows the critical role of stem cells in the biology of IH (11, 27, 89, 90) with emerging evidence suggesting an embryonic developmental anomaly due to aberrant proliferation and differentiation of a hemogenic endothelium (12) with a neural crest phenotype (32), that possesses the capacity for endothelial (13), hematopoietic (15), mesenchymal (14), and neuronal (9) differentiation (Figure 6). Current evidence suggests a putative PCVMCCs embolic origin of IH during the first trimester (10) (Figure 6), although this remains to be conclusively elucidated. The timing of embolization of PCVMCCs to the fetus in relation to the timing of neural crest cell migration down their somitic migration routes, may account for the morphology (discrete or segmental) of the lesions (32, 91).

There is strong evidence of the involvement of the VEGFR-2 and its ligand VEGF-A (46, 48, 51) and the IGF-2 ligand in promoting cellular proliferation (42, 55), and the TRAIL-OPG anti-apoptotic pathway in preventing cellular apoptosis (58) in IH (Figure 6).

The discovery of the role of the RAS in the biology of IH (25) provides a plausible explanation for the programed biologic behavior and the β-blocker-induced accelerated involution of this tumor (25) (Figure 6). The observation that the vasoactive peptide, ATII, promotes cellular proliferation in IH predominantly via its action on the ATIIR2 isoform (63) is significant. The role of the RAS in the biology of IH is further supported by the effect of captopril, an ACE inhibitor, in inducing accelerated involution of IH (66) (Figure 6).

The discovery of the critical role of RAS in IH represents a novel and fascinating paradigm shift in the understanding of human development, IH, and other tumors in general (92).

## Conflict of Interest Statement

The authors declare that the research was conducted in the absence of any commercial or financial relationships that could be construed as a potential conflict of interest.
